# {5,5′-Dimeth­oxy-2,2′-[1,1′-(2,2-dimethyl­propane-1,3-diyldinitrilo)­diethyl­idyne]diphenolato-κ^4^ *O*,*N*,*N*′,*O*′}copper(II) monohydrate

**DOI:** 10.1107/S160053681103889X

**Published:** 2011-09-30

**Authors:** Akbar Ghaemi, Saeed Rayati, Ehsan Elahi, Seik Weng Ng, Edward R. T. Tiekink

**Affiliations:** a Department of Chemistry, Saveh Branch, Islamic Azad University, Saveh, Iran; b Department of Chemistry, K. N. Toosi University of Technology, PO Box, 16315-1618, Tehran, Iran; c Department of Chemistry, University of Malaya, 50603 Kuala Lumpur, Malaysia; d Chemistry Department, Faculty of Science, King Abdulaziz University, PO Box 80203 Jeddah, Saudi Arabia

## Abstract

The tetra­dentate dianion in the title complex hydrate, [Cu(C_23_H_28_N_2_O_4_)]·H_2_O, provides the Cu^II^ atom with a *cis*-N_2_O_2_ donor set. There is a significant twist from a regular square-planar geometry with the dihedral angle formed between the two six-membered CuOC_3_N chelate rings being 32.14 (8)°. The water mol­ecule forms hydrogen bonds to each of the coordinating O atoms of a given complex mol­ecule. Supra­molecular layers in the *bc* plane are formed in the crystal packing through C—H⋯O and C—H⋯π inter­actions.

## Related literature

For the catalytic potential of Schiff base complexes of Cu^II^, see: Gupta & Sutar (2008 ▶); Rayati *et al.* (2010 ▶). For the structure of the ligand, see: Ghaemi *et al.* (2011 ▶). For crystallization conditions, see: Harrowfield *et al.* (1996 ▶).
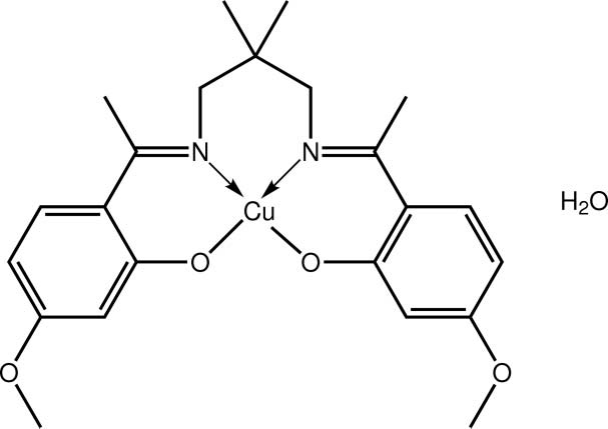


## Experimental

### 

#### Crystal data

[Cu(C_23_H_28_N_2_O_4_)]·H_2_O
*M* *_r_* = 478.03
Triclinic, 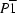

*a* = 10.4721 (7) Å
*b* = 10.8023 (9) Å
*c* = 10.8487 (7) Å
α = 106.699 (7)°
β = 99.823 (5)°
γ = 100.035 (6)°
*V* = 1125.37 (14) Å^3^
*Z* = 2
Mo *K*α radiation
μ = 1.01 mm^−1^
*T* = 294 K
0.40 × 0.40 × 0.20 mm

#### Data collection

Agilent SuperNova Dual diffractometer with Atlas detector
Absorption correction: multi-scan (*CrysAlis PRO*; Agilent, 2010 ▶) *T*_min_ = 0.643, *T*_max_ = 1.000
11143 measured reflections
5034 independent reflections
4332 reflections with *I* > 2σ(*I*)
*R*_int_ = 0.024

#### Refinement

*R*[*F* ^2^ > 2σ(*F* ^2^)] = 0.037
*wR*(*F* ^2^) = 0.105
*S* = 0.99
5034 reflections
285 parameters
6 restraints
H-atom parameters constrained
Δρ_max_ = 0.26 e Å^−3^
Δρ_min_ = −0.47 e Å^−3^

### 

Data collection: *CrysAlis PRO* (Agilent, 2010 ▶); cell refinement: *CrysAlis PRO*; data reduction: *CrysAlis PRO*; program(s) used to solve structure: *SHELXS97* (Sheldrick, 2008 ▶); program(s) used to refine structure: *SHELXL97* (Sheldrick, 2008 ▶); molecular graphics: *ORTEP-3* (Farrugia, 1997 ▶) and *DIAMOND* (Brandenburg, 2006 ▶); software used to prepare material for publication: *publCIF* (Westrip, 2010 ▶).

## Supplementary Material

Crystal structure: contains datablock(s) general, I. DOI: 10.1107/S160053681103889X/hg5100sup1.cif

Structure factors: contains datablock(s) I. DOI: 10.1107/S160053681103889X/hg5100Isup2.hkl

Additional supplementary materials:  crystallographic information; 3D view; checkCIF report

## Figures and Tables

**Table 1:** Selected bond lengths (Å)

Cu—O2	1.8825 (16)
Cu—O3	1.8776 (15)
Cu—N1	1.9597 (17)
Cu—N2	1.9524 (18)

**Table 2:** Hydrogen-bond geometry (Å, °)

*D*—H⋯*A*	*D*—H	H⋯*A*	*D*⋯*A*	*D*—H⋯*A*
O1*w*—H1*w*⋯O2	0.84	2.12	2.832 (3)	142
O1*w*—H2*w*⋯O3	0.84	2.32	3.035 (3)	143
C7—H7c⋯O1*w*^i^	0.96	2.55	3.476 (5)	163
C16—H16c⋯O2^ii^	0.96	2.52	3.409 (3)	153
C14—H14b⋯*Cg*1^ii^	0.97	2.62	3.426 (2)	141

Symmetry codes: (i) 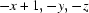; (ii) 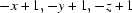.

**Fig. 1.**
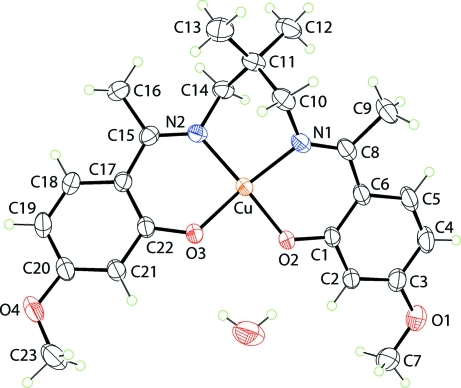
The molecular structure of (I) showing the atom-labelling scheme and displacement ellipsoids at the 50% probability level.

**Fig. 2.**
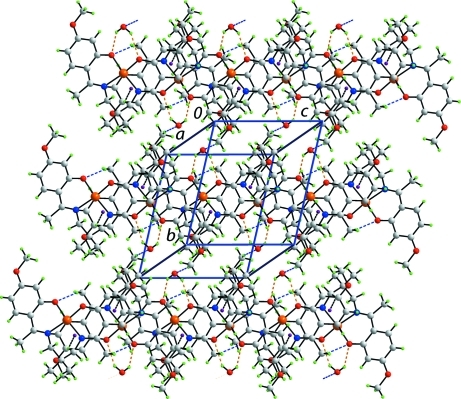
Supramolecular layer in the bc plane in (I) sustained by C—H···O and C—H···π interactions shown as blue and black dashed lines, respectively. The O—H···O hydrogen bonds are shown as orange dashed lines.

**Fig. 3.**
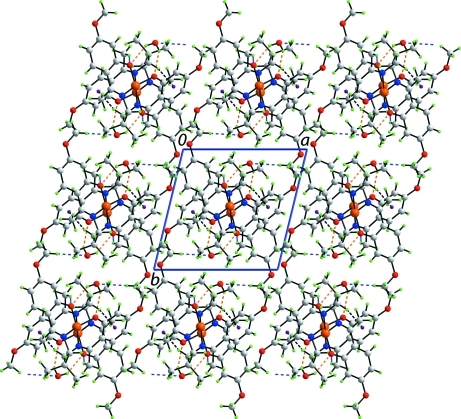
A view in projection down the c axis of the unit-cell contents of (I), highlighting the stacking of layers along the a axis. The C—H···O and C—H···π interactions shown as blue and black dashed lines, respectively, and the O—H···O hydrogen bonds are shown as orange dashed lines.

## supplementary crystallographic information

### Comment

Synthetic copper(II) Schiff base complexes have long been of great interest because of their potential as catalysts in the oxidation of various organic compounds (Gupta & Sutar, 2008). In continuation of research in this field (Rayati *et al.*, 2010), the title complex, (I), was investigated.

The tetradentate dianion in the title monohydrate, (I), Fig. 1, provides a *cis*-N_2_O_2_ donor set, Table 1. Three six-membered chelate rings are formed as a result of coordination of the dianion. The CuNC_3_N ring adopts a half-chair conformation. While the CuOC_3_N chelate ring containing the O3 atom approaches planarity with a r.m.s. deviation of 0.031 Å, the other ring displays significant distortions. Thus, the r.m.s. deviation for the O2-containing CuOC_3_N chelate ring is 0.163 Å with maximum deviations of 0.162 (2) Å for atom O2 and -0.159 (1) Å for the Cu atom. The dihedral angle formed between the two CuOC_3_N chelate rings is 32.14 (8)° indicating a significant distortion from a regular square planar geometry. Each of the methoxy groups is co-planar with the benzene ring to which it is attached as seen in the values of the C7—O1—C3—C2 and C23—O4—C20—C19 of -0.8 (4) and -179.3 (3)°, respectively. The water molecule of solvation is associated with the complex, forming a bridge *via* its hydrogen atoms between the two coordinated oxygen atoms, Table 2.

The crystal packing features C—H···O and C—H···π interactions, Table 2, that assemble molecules into layers in the *bc* plane, Fig. 2, which stack along the *a* axis, Fig. 3.

### Experimental

The title complex was obtained by the template method in a branch tube (Harrowfield *et al.*, 1996). The recently described (Ghaemi *et al.*, 2011) *N*,*N*'-bis(2-hydroxy-4-methoxyacetophenone)-2,2-dimethylpropane-1,3-diamine (0.40 g, 1 mmol) and copper(II) acetate monohydrate (0.199 g, 1 mmol) were placed in the main arm of a branched tube. Ethanol was added to fill both arms. The tube was sealed and the main arm immersed in an oil bath at 333 K while the other was held at ambient temperature. After one week, crystals deposited in the cooler arm. These were filtered off and air dried. Yield: 75%. FT—IR data: ν(C═N) 1595 cm^-1^.

### Refinement

The H-atoms were placed in calculated positions (C—H 0.93 to 0.97 Å) and were included in the refinement in the riding model approximation, with *U*_iso_(H) set to 1.2 to 1.5*U*_equiv_(C). The water-H atoms were placed in calculated positions (O—H = 0.84 Å; 1.5*U*_equiv_(O) on the basis of hydrogen bonding.

### Crystal data

**Table:**

[Cu(C_23_H_28_N_2_O_4_)]·H_2_O	*Z* = 2
*M**_r_* = 478.03	*F*(000) = 502
Triclinic, *P*1	*D*_x_ = 1.411 Mg m^−^^3^
Hall symbol: -P 1	Mo *K*α radiation, λ = 0.71073 Å
*a* = 10.4721 (7) Å	Cell parameters from 5694 reflections
*b* = 10.8023 (9) Å	θ = 2.3–29.3°
*c* = 10.8487 (7) Å	µ = 1.01 mm^−^^1^
α = 106.699 (7)°	*T* = 294 K
β = 99.823 (5)°	Block, dark-brown
γ = 100.035 (6)°	0.40 × 0.40 × 0.20 mm
*V* = 1125.37 (14) Å^3^	

### Data collection

**Table:**

Agilent SuperNova Dual diffractometer with Atlas detector	5034 independent reflections
Radiation source: SuperNova (Mo) X-ray Source	4332 reflections with *I* > 2σ(*I*)
Mirror	*R*_int_ = 0.024
Detector resolution: 10.4041 pixels mm^-1^	θ_max_ = 27.5°, θ_min_ = 2.5°
ω scan	*h* = −12→13
Absorption correction: multi-scan (*CrysAlis PRO*; Agilent, 2010)	*k* = −14→13
*T*_min_ = 0.643, *T*_max_ = 1.000	*l* = −14→11
11143 measured reflections	

### Refinement

**Table:**

Refinement on *F*^2^	Primary atom site location: structure-invariant direct methods
Least-squares matrix: full	Secondary atom site location: difference Fourier map
*R*[*F*^2^ > 2σ(*F*^2^)] = 0.037	Hydrogen site location: inferred from neighbouring sites
*wR*(*F*^2^) = 0.105	H-atom parameters constrained
*S* = 0.99	*w* = 1/[σ^2^(*F*_o_^2^) + (0.0531*P*)^2^ + 0.3861*P*] where *P* = (*F*_o_^2^ + 2*F*_c_^2^)/3
5034 reflections	(Δ/σ)_max_ = 0.002
285 parameters	Δρ_max_ = 0.26 e Å^−^^3^
6 restraints	Δρ_min_ = −0.47 e Å^−^^3^

### Special details

**Table:**

Geometry. All e.s.d.'s (except the e.s.d. in the dihedral angle between two l.s. planes) are estimated using the full covariance matrix. The cell e.s.d.'s are taken into account individually in the estimation of e.s.d.'s in distances, angles and torsion angles; correlations between e.s.d.'s in cell parameters are only used when they are defined by crystal symmetry. An approximate (isotropic) treatment of cell e.s.d.'s is used for estimating e.s.d.'s involving l.s. planes.
Refinement. Refinement of *F*^2^ against ALL reflections. The weighted *R*-factor *wR* and goodness of fit *S* are based on *F*^2^, conventional *R*-factors *R* are based on *F*, with *F* set to zero for negative *F*^2^. The threshold expression of *F*^2^ > σ(*F*^2^) is used only for calculating *R*-factors(gt) *etc*. and is not relevant to the choice of reflections for refinement. *R*-factors based on *F*^2^ are statistically about twice as large as those based on *F*, and *R*- factors based on ALL data will be even larger.

### Fractional atomic coordinates and isotropic or equivalent isotropic displacement parameters (Å^2^)

**Table:**

	*x*	*y*	*z*	*U*_iso_*/*U*_eq_	
Cu	0.48963 (3)	0.47337 (3)	0.24182 (2)	0.04175 (11)	
O1	0.04084 (19)	−0.0426 (2)	−0.1635 (2)	0.0746 (6)	
O2	0.40205 (16)	0.30214 (16)	0.12419 (15)	0.0519 (4)	
O3	0.62185 (18)	0.39699 (17)	0.30882 (16)	0.0580 (5)	
O4	0.96607 (19)	0.3235 (2)	0.60237 (19)	0.0702 (5)	
O1w	0.5646 (3)	0.1173 (3)	0.1144 (3)	0.1143 (10)	
H1w	0.4956	0.1403	0.0867	0.171*	
H2w	0.6121	0.1815	0.1794	0.171*	
N1	0.40191 (19)	0.55577 (19)	0.12292 (18)	0.0440 (4)	
N2	0.54285 (18)	0.63414 (17)	0.39652 (18)	0.0404 (4)	
C1	0.2881 (2)	0.2697 (2)	0.0363 (2)	0.0429 (5)	
C2	0.2249 (2)	0.1342 (2)	−0.0152 (2)	0.0472 (5)	
H2	0.2638	0.0743	0.0155	0.057*	
C3	0.1068 (2)	0.0873 (3)	−0.1098 (2)	0.0553 (6)	
C4	0.0476 (3)	0.1766 (3)	−0.1551 (3)	0.0685 (8)	
H4	−0.0329	0.1462	−0.2182	0.082*	
C5	0.1076 (3)	0.3079 (3)	−0.1070 (3)	0.0607 (7)	
H5	0.0664	0.3657	−0.1392	0.073*	
C6	0.2299 (2)	0.3623 (2)	−0.0100 (2)	0.0461 (5)	
C7	0.0998 (3)	−0.1364 (3)	−0.1189 (3)	0.0780 (9)	
H7A	0.0449	−0.2245	−0.1647	0.117*	
H7B	0.1071	−0.1161	−0.0255	0.117*	
H7C	0.1869	−0.1317	−0.1367	0.117*	
C8	0.2965 (2)	0.5025 (3)	0.0267 (2)	0.0481 (6)	
C9	0.2384 (3)	0.5809 (3)	−0.0542 (3)	0.0696 (8)	
H9A	0.2902	0.6711	−0.0212	0.104*	
H9B	0.1480	0.5795	−0.0475	0.104*	
H9C	0.2401	0.5420	−0.1452	0.104*	
C10	0.4828 (3)	0.6911 (2)	0.1535 (2)	0.0520 (6)	
H10A	0.4573	0.7230	0.0801	0.062*	
H10B	0.5759	0.6882	0.1623	0.062*	
C11	0.4675 (3)	0.7898 (2)	0.2811 (3)	0.0511 (6)	
C12	0.3407 (3)	0.8406 (3)	0.2542 (3)	0.0714 (8)	
H12A	0.3482	0.8891	0.1932	0.107*	
H12B	0.3301	0.8980	0.3358	0.107*	
H12C	0.2646	0.7665	0.2170	0.107*	
C13	0.5910 (3)	0.9054 (3)	0.3299 (3)	0.0732 (8)	
H13A	0.5994	0.9441	0.2615	0.110*	
H13B	0.6688	0.8732	0.3519	0.110*	
H13C	0.5822	0.9713	0.4070	0.110*	
C14	0.4513 (2)	0.7208 (2)	0.3848 (2)	0.0456 (5)	
H14A	0.4652	0.7883	0.4703	0.055*	
H14B	0.3604	0.6681	0.3623	0.055*	
C15	0.6363 (2)	0.6611 (2)	0.5039 (2)	0.0434 (5)	
C16	0.6651 (3)	0.7919 (2)	0.6149 (3)	0.0632 (7)	
H16A	0.6322	0.8562	0.5812	0.095*	
H16B	0.7596	0.8230	0.6508	0.095*	
H16C	0.6218	0.7794	0.6831	0.095*	
C17	0.7159 (2)	0.5667 (2)	0.5215 (2)	0.0423 (5)	
C18	0.8061 (3)	0.5949 (3)	0.6445 (2)	0.0564 (6)	
H18	0.8115	0.6730	0.7120	0.068*	
C19	0.8854 (3)	0.5134 (3)	0.6686 (3)	0.0635 (7)	
H19	0.9418	0.5351	0.7516	0.076*	
C20	0.8819 (2)	0.3976 (3)	0.5691 (2)	0.0508 (6)	
C21	0.7941 (2)	0.3632 (2)	0.4486 (2)	0.0459 (5)	
H21	0.7917	0.2854	0.3822	0.055*	
C22	0.7074 (2)	0.4443 (2)	0.4242 (2)	0.0425 (5)	
C23	0.9685 (3)	0.2051 (4)	0.5051 (3)	0.0800 (9)	
H23A	1.0308	0.1625	0.5422	0.120*	
H23B	0.9951	0.2259	0.4315	0.120*	
H23C	0.8812	0.1465	0.4754	0.120*	

### Atomic displacement parameters (Å^2^)

**Table:**

	*U*^11^	*U*^22^	*U*^33^	*U*^12^	*U*^13^	*U*^23^
Cu	0.04730 (18)	0.04062 (18)	0.03637 (16)	0.01857 (13)	0.00166 (12)	0.01100 (12)
O1	0.0582 (11)	0.0691 (13)	0.0700 (12)	0.0033 (10)	−0.0159 (10)	0.0074 (10)
O2	0.0550 (9)	0.0437 (9)	0.0456 (9)	0.0209 (8)	−0.0136 (7)	0.0063 (7)
O3	0.0669 (11)	0.0539 (10)	0.0417 (8)	0.0319 (9)	−0.0107 (8)	0.0014 (7)
O4	0.0647 (12)	0.0861 (14)	0.0622 (11)	0.0343 (11)	−0.0056 (9)	0.0297 (11)
O1w	0.0964 (18)	0.0788 (16)	0.153 (2)	0.0434 (14)	−0.0017 (17)	0.0207 (17)
N1	0.0504 (10)	0.0491 (11)	0.0414 (9)	0.0236 (9)	0.0130 (8)	0.0198 (8)
N2	0.0443 (10)	0.0368 (9)	0.0434 (9)	0.0130 (8)	0.0108 (8)	0.0154 (8)
C1	0.0449 (12)	0.0550 (13)	0.0294 (9)	0.0224 (10)	0.0044 (9)	0.0110 (9)
C2	0.0471 (12)	0.0536 (14)	0.0371 (11)	0.0195 (11)	0.0007 (9)	0.0099 (10)
C3	0.0486 (13)	0.0643 (16)	0.0437 (12)	0.0144 (12)	0.0010 (10)	0.0087 (12)
C4	0.0521 (15)	0.087 (2)	0.0535 (15)	0.0173 (15)	−0.0137 (12)	0.0185 (15)
C5	0.0559 (15)	0.0807 (19)	0.0504 (14)	0.0302 (14)	−0.0010 (12)	0.0283 (14)
C6	0.0480 (12)	0.0607 (15)	0.0348 (10)	0.0256 (11)	0.0063 (9)	0.0178 (10)
C7	0.0662 (18)	0.0586 (17)	0.086 (2)	0.0091 (15)	−0.0071 (16)	0.0059 (16)
C8	0.0545 (13)	0.0627 (15)	0.0400 (11)	0.0308 (12)	0.0133 (10)	0.0251 (11)
C9	0.0762 (19)	0.079 (2)	0.0666 (17)	0.0324 (16)	0.0040 (14)	0.0419 (16)
C10	0.0565 (14)	0.0581 (15)	0.0553 (14)	0.0217 (12)	0.0194 (11)	0.0313 (12)
C11	0.0610 (14)	0.0421 (12)	0.0596 (14)	0.0211 (11)	0.0165 (12)	0.0242 (11)
C12	0.087 (2)	0.0629 (17)	0.0770 (19)	0.0444 (16)	0.0179 (16)	0.0274 (15)
C13	0.084 (2)	0.0560 (16)	0.081 (2)	0.0073 (15)	0.0165 (17)	0.0320 (15)
C14	0.0522 (13)	0.0409 (12)	0.0482 (12)	0.0187 (10)	0.0169 (10)	0.0136 (10)
C15	0.0487 (12)	0.0373 (11)	0.0406 (11)	0.0041 (10)	0.0098 (10)	0.0113 (9)
C16	0.086 (2)	0.0412 (13)	0.0514 (14)	0.0139 (13)	0.0024 (13)	0.0066 (11)
C17	0.0414 (11)	0.0394 (11)	0.0411 (11)	0.0037 (9)	0.0021 (9)	0.0136 (9)
C18	0.0568 (14)	0.0482 (14)	0.0470 (13)	0.0036 (12)	−0.0092 (11)	0.0069 (11)
C19	0.0554 (15)	0.0667 (17)	0.0531 (14)	0.0070 (13)	−0.0161 (12)	0.0173 (13)
C20	0.0406 (12)	0.0599 (15)	0.0524 (13)	0.0119 (11)	0.0003 (10)	0.0252 (12)
C21	0.0434 (12)	0.0536 (14)	0.0422 (11)	0.0174 (10)	0.0060 (9)	0.0169 (10)
C22	0.0400 (11)	0.0478 (12)	0.0378 (11)	0.0098 (10)	0.0018 (9)	0.0157 (9)
C23	0.082 (2)	0.099 (2)	0.077 (2)	0.056 (2)	0.0146 (17)	0.0381 (19)

### Geometric parameters (Å, °)

**Table:**

Cu—O2	1.8825 (16)	C9—H9C	0.9600
Cu—O3	1.8776 (15)	C10—C11	1.538 (3)
Cu—N1	1.9597 (17)	C10—H10A	0.9700
Cu—N2	1.9524 (18)	C10—H10B	0.9700
O1—C3	1.358 (3)	C11—C13	1.528 (4)
O1—C7	1.428 (3)	C11—C14	1.535 (3)
O2—C1	1.316 (3)	C11—C12	1.538 (3)
O3—C22	1.312 (3)	C12—H12A	0.9600
O4—C20	1.361 (3)	C12—H12B	0.9600
O4—C23	1.412 (4)	C12—H12C	0.9600
O1w—H1w	0.8400	C13—H13A	0.9600
O1w—H2w	0.8400	C13—H13B	0.9600
N1—C8	1.296 (3)	C13—H13C	0.9600
N1—C10	1.469 (3)	C14—H14A	0.9700
N2—C15	1.310 (3)	C14—H14B	0.9700
N2—C14	1.466 (3)	C15—C17	1.458 (3)
C1—C2	1.400 (3)	C15—C16	1.512 (3)
C1—C6	1.421 (3)	C16—H16A	0.9600
C2—C3	1.374 (3)	C16—H16B	0.9600
C2—H2	0.9300	C16—H16C	0.9600
C3—C4	1.390 (4)	C17—C22	1.412 (3)
C4—C5	1.353 (4)	C17—C18	1.414 (3)
C4—H4	0.9300	C18—C19	1.359 (4)
C5—C6	1.420 (3)	C18—H18	0.9300
C5—H5	0.9300	C19—C20	1.387 (4)
C6—C8	1.459 (4)	C19—H19	0.9300
C7—H7A	0.9600	C20—C21	1.373 (3)
C7—H7B	0.9600	C21—C22	1.411 (3)
C7—H7C	0.9600	C21—H21	0.9300
C8—C9	1.511 (3)	C23—H23A	0.9600
C9—H9A	0.9600	C23—H23B	0.9600
C9—H9B	0.9600	C23—H23C	0.9600
			
O3—Cu—O2	87.70 (7)	C13—C11—C10	107.4 (2)
O3—Cu—N2	93.30 (7)	C14—C11—C10	110.41 (18)
O2—Cu—N2	161.99 (8)	C13—C11—C12	110.3 (2)
O3—Cu—N1	156.09 (8)	C14—C11—C12	106.3 (2)
O2—Cu—N1	91.13 (7)	C10—C11—C12	110.7 (2)
N2—Cu—N1	95.08 (8)	C11—C12—H12A	109.5
C3—O1—C7	117.2 (2)	C11—C12—H12B	109.5
C1—O2—Cu	126.53 (14)	H12A—C12—H12B	109.5
C22—O3—Cu	128.03 (15)	C11—C12—H12C	109.5
C20—O4—C23	118.3 (2)	H12A—C12—H12C	109.5
H1w—O1w—H2w	107.4	H12B—C12—H12C	109.5
C8—N1—C10	123.47 (19)	C11—C13—H13A	109.5
C8—N1—Cu	128.32 (17)	C11—C13—H13B	109.5
C10—N1—Cu	108.06 (14)	H13A—C13—H13B	109.5
C15—N2—C14	121.92 (19)	C11—C13—H13C	109.5
C15—N2—Cu	127.82 (15)	H13A—C13—H13C	109.5
C14—N2—Cu	109.93 (14)	H13B—C13—H13C	109.5
O2—C1—C2	116.13 (19)	N2—C14—C11	114.24 (18)
O2—C1—C6	124.1 (2)	N2—C14—H14A	108.7
C2—C1—C6	119.7 (2)	C11—C14—H14A	108.7
C3—C2—C1	121.7 (2)	N2—C14—H14B	108.7
C3—C2—H2	119.1	C11—C14—H14B	108.7
C1—C2—H2	119.1	H14A—C14—H14B	107.6
O1—C3—C2	124.5 (2)	N2—C15—C17	121.6 (2)
O1—C3—C4	116.1 (2)	N2—C15—C16	120.9 (2)
C2—C3—C4	119.4 (3)	C17—C15—C16	117.5 (2)
C5—C4—C3	119.7 (2)	C15—C16—H16A	109.5
C5—C4—H4	120.1	C15—C16—H16B	109.5
C3—C4—H4	120.1	H16A—C16—H16B	109.5
C4—C5—C6	123.6 (2)	C15—C16—H16C	109.5
C4—C5—H5	118.2	H16A—C16—H16C	109.5
C6—C5—H5	118.2	H16B—C16—H16C	109.5
C5—C6—C1	115.8 (2)	C22—C17—C18	116.3 (2)
C5—C6—C8	120.7 (2)	C22—C17—C15	124.43 (19)
C1—C6—C8	123.2 (2)	C18—C17—C15	119.3 (2)
O1—C7—H7A	109.5	C19—C18—C17	123.1 (2)
O1—C7—H7B	109.5	C19—C18—H18	118.5
H7A—C7—H7B	109.5	C17—C18—H18	118.5
O1—C7—H7C	109.5	C18—C19—C20	119.9 (2)
H7A—C7—H7C	109.5	C18—C19—H19	120.1
H7B—C7—H7C	109.5	C20—C19—H19	120.1
N1—C8—C6	121.1 (2)	O4—C20—C21	124.7 (2)
N1—C8—C9	122.0 (2)	O4—C20—C19	115.5 (2)
C6—C8—C9	116.9 (2)	C21—C20—C19	119.7 (2)
C8—C9—H9A	109.4	C20—C21—C22	120.9 (2)
C8—C9—H9B	109.4	C20—C21—H21	119.6
H9A—C9—H9B	109.5	C22—C21—H21	119.6
C8—C9—H9C	109.6	O3—C22—C21	115.4 (2)
H9A—C9—H9C	109.5	O3—C22—C17	124.6 (2)
H9B—C9—H9C	109.5	C21—C22—C17	119.95 (19)
N1—C10—C11	113.23 (19)	O4—C23—H23A	109.5
N1—C10—H10A	108.9	O4—C23—H23B	109.5
C11—C10—H10A	108.9	H23A—C23—H23B	109.5
N1—C10—H10B	108.9	O4—C23—H23C	109.5
C11—C10—H10B	108.9	H23A—C23—H23C	109.5
H10A—C10—H10B	107.7	H23B—C23—H23C	109.5
C13—C11—C14	111.8 (2)		
			
O3—Cu—O2—C1	178.8 (2)	C5—C6—C8—N1	174.0 (2)
N2—Cu—O2—C1	85.3 (3)	C1—C6—C8—N1	−13.1 (3)
N1—Cu—O2—C1	−25.1 (2)	C5—C6—C8—C9	−7.1 (3)
O2—Cu—O3—C22	−158.8 (2)	C1—C6—C8—C9	165.8 (2)
N2—Cu—O3—C22	3.2 (2)	C8—N1—C10—C11	108.3 (3)
N1—Cu—O3—C22	113.6 (2)	Cu—N1—C10—C11	−76.0 (2)
O3—Cu—N1—C8	104.7 (3)	N1—C10—C11—C13	158.1 (2)
O2—Cu—N1—C8	17.8 (2)	N1—C10—C11—C14	36.0 (3)
N2—Cu—N1—C8	−145.2 (2)	N1—C10—C11—C12	−81.4 (2)
O3—Cu—N1—C10	−70.8 (2)	C15—N2—C14—C11	113.8 (2)
O2—Cu—N1—C10	−157.67 (15)	Cu—N2—C14—C11	−72.3 (2)
N2—Cu—N1—C10	39.26 (15)	C13—C11—C14—N2	−75.8 (3)
O3—Cu—N2—C15	−2.6 (2)	C10—C11—C14—N2	43.7 (3)
O2—Cu—N2—C15	90.1 (3)	C12—C11—C14—N2	163.8 (2)
N1—Cu—N2—C15	−160.20 (19)	C14—N2—C15—C17	172.1 (2)
O3—Cu—N2—C14	−176.04 (14)	Cu—N2—C15—C17	−0.6 (3)
O2—Cu—N2—C14	−83.4 (2)	C14—N2—C15—C16	−7.6 (3)
N1—Cu—N2—C14	26.38 (15)	Cu—N2—C15—C16	179.70 (18)
Cu—O2—C1—C2	−163.66 (16)	N2—C15—C17—C22	4.5 (4)
Cu—O2—C1—C6	18.1 (3)	C16—C15—C17—C22	−175.7 (2)
O2—C1—C2—C3	−178.5 (2)	N2—C15—C17—C18	−173.7 (2)
C6—C1—C2—C3	−0.2 (3)	C16—C15—C17—C18	6.0 (3)
C7—O1—C3—C2	−0.8 (4)	C22—C17—C18—C19	2.6 (4)
C7—O1—C3—C4	179.8 (3)	C15—C17—C18—C19	−179.0 (2)
C1—C2—C3—O1	−180.0 (2)	C17—C18—C19—C20	1.4 (4)
C1—C2—C3—C4	−0.6 (4)	C23—O4—C20—C21	2.8 (4)
O1—C3—C4—C5	−179.7 (3)	C23—O4—C20—C19	−179.3 (3)
C2—C3—C4—C5	0.9 (4)	C18—C19—C20—O4	179.2 (2)
C3—C4—C5—C6	−0.4 (5)	C18—C19—C20—C21	−2.9 (4)
C4—C5—C6—C1	−0.3 (4)	O4—C20—C21—C22	178.0 (2)
C4—C5—C6—C8	173.1 (3)	C19—C20—C21—C22	0.3 (4)
O2—C1—C6—C5	178.8 (2)	Cu—O3—C22—C21	178.78 (16)
C2—C1—C6—C5	0.7 (3)	Cu—O3—C22—C17	−0.6 (4)
O2—C1—C6—C8	5.6 (3)	C20—C21—C22—O3	−175.6 (2)
C2—C1—C6—C8	−172.6 (2)	C20—C21—C22—C17	3.9 (4)
C10—N1—C8—C6	172.0 (2)	C18—C17—C22—O3	174.3 (2)
Cu—N1—C8—C6	−2.9 (3)	C15—C17—C22—O3	−4.0 (4)
C10—N1—C8—C9	−6.9 (4)	C18—C17—C22—C21	−5.1 (3)
Cu—N1—C8—C9	178.26 (18)	C15—C17—C22—C21	176.6 (2)

### Hydrogen-bond geometry (Å, °)

**Table:**

*D*—H···*A*	*D*—H	H···*A*	*D*···*A*	*D*—H···*A*
O1w—H1w···O2	0.84	2.12	2.832 (3)	142
O1w—H2w···O3	0.84	2.32	3.035 (3)	143
C7—H7c···O1w^i^	0.96	2.55	3.476 (5)	163
C16—H16c···O2^ii^	0.96	2.52	3.409 (3)	153
C14—H14b···Cg1^ii^	0.97	2.62	3.426 (2)	141

Symmetry codes: (i) −*x*+1, −*y*, −*z*; (ii) −*x*+1, −*y*+1, −*z*+1.
